# Supplementary material for: Cross-coupling polycondensation via C–O or C–N bond cleavage
Source: Nat Commun. 2018 Apr 23;9:1587. doi: 10.1038/s41467-018-03928-z (PMC5913252; doi:10.1038/s41467-018-03928-z)
Supplement: Supplementary file 1 — Supplementary Information [file 41467_2018_3928_MOESM1_ESM.pdf]

# Supplementary Information

# Cross-Coupling Polycondensation *via* C–O or C–N Bond Cleavage

Ze-Kun Yang,[1,2] Ning-Xin Xu,[1] Ryo Takita,[2] Atsuya Muranaka,[2] Chao Wang,*[1,2] and Masanobu Uchiyama.*[1,2]

[1] Graduate School of Pharmaceutical Sciences, University of Tokyo, 7-3-1 Hongo, Bunkyo-ku, Tokyo 113-0033, Japan;
[2] Advanced Elements Chemistry Research Team, Center for Sustainable Resource Science, and Elements Chemistry Laboratory, RIKEN, 2-1 Hirosawa, Wako-shi, Saitama 351-0198, Japan.

*E-mail: chaowang@mol.f.u-tokyo.ac.jp (C.W.); uchiyama@mol.f.u-tokyo.ac.jp (M.U.)

# Supplementary Methods

## General Methods

All reactions were carried out under a slightly positive pressure of dry argon by using standard Schlenk line techniques or in Glovebox (Braun, Labmaster SP). The oxygen and moisture concentrations in the glovebox atmosphere were monitored by the $O_2/H_2O$ analyzer to ensure both were always below 0.1 ppm. Unless otherwise noted, all starting materials including dehydrated solvents were purchased from Wako, Kanto, TCI, or Aldrich. Polymer molecular weights were determined by comparison with polystyrene standards using TOSOH HLC-8020GPC Gel-Permeation Chromatography equipped with two Shodex GPC KF-G columns in sequence and analyzed with TOSOH UV-8020 absorbance detector (254 nm). Samples were dissolved in THF and passed through a 0.2 µm PTFE filter prior to analysis. Circular dichroism (CD) spectra were measured using a JASCO J-820 CD spectropolarimeter equipped with a programmed temperature-controller (JASCO, Tokyo, Japan), and the solvent of sample was dichloromethane. NMR spectra were recorded on a Bruker AVANCE III 500 spectrometer (FT, 500 MHz for $^1$H). Chemical shift values are reported in ppm with the solvent resonance as the internal standard ($CHCl_3$: δ 7.26 for $^1$H). **Attention:** All organometallics (Mg, Li) should be handled with care, as many are pyrophoric or extremely sensitive to oxygen/moisture.

## Preparation of Substrates

**General procedure for preparation of Grignard reagents 1a:**

**Method A:** Under an argon atmosphere, a mixture of freshly cut magnesium turnings (0.972 g, 40 mmol) and 20 mL of dry THF in an oven-dried flask was stirred and a solution of 2,7-dibromide-9,9-dihexylfluorine (**0a**) (1.968 g, 4 mmol) in 5 mL of dry THF was added slowly through a syringe. The solution was allowed to stir at reflux for 12 h and the obtained brown transparent solution was titrated with $Te_2Ph_2$ [see: Aso, Y. *et al.* Simple titration method using diphenyl ditelluride as a colored indicator for the determination of organolithium and organomagnesium reagents. *J. Org. Chem.* **54,** 5627–5629 (1989)] and kept carefully at room temperature.

**Method B:** Under an argon atmosphere, a mixture of freshly cut magnesium turnings (0.972 g, 40 mmol) and 20 mL of dry THF in an oven-dried flask was stirred and 1,2-dibromoethane (7.5 mg, 0.04 mmol) was added. The mixture was heated to 50 °C over a period of 10 min, and a solution of 2,7-dibromide-9,9-dihexylfluorine (**0a**) (1.968 g, 4 mmol) in 5 mL of dry THF was added slowly through a syringe. The solution was allowed to stir at 50 °C for 1 h and the obtained brown transparent solution was titrated with $Te_2Ph_2$ and kept carefully at 4 °C.

**Method C:** In glovebox, a 50 mL Schlenk flask equipped with a Teflon-coated magnetic stirring bar was charged with freshly cut lithium (0.278 g, 40 mmol), anhydrous magnesium chloride (2.095 g, 22 mmol) and naphthalene (0.512 g, 4 mmol), and 20 mL of dry THF. Then the flask was taken out, the mixture was stirred vigorously with ultrasonic stirrer. A black precipitate started to form within a few minutes. The solution was allowed to stir at room temperature for 12 h, producing Rieke magnesium as a grey powder that settles slowly when the stirring is stopped. The Schlenk flask was moved into the glovebox and the Rieke magnesium was transferred to another Schlenk flask via cannula to separate from unreacted lithium. After about 20 minutes' standing, the supernatant was removed and 10 mL dry THF was added to the Rieke magnesium, repeated this operation until the THF is colorless, and the Rieke magnesium was ready for next usage.

Under an argon atmosphere, a mixture of Rieke magnesium (about 0.480 g, 20 mmol) and 10 mL of dry THF in an oven-dried Schlenk flask was stirred at -78 °C, and a solution of 2,7-dibromide-9,9-dihexylfluorine (**0a**) (1.968 g, 4 mmol) in 5 mL of dry THF was added slowly through a syringe. The solution was allowed to stir at -78 °C for 1 h and the obtained grey muddy solution was transferred into the centrifuge tubes in glovebox. The centrifuge tubes were carefully sealed and centrifuged at 3000 rpm for 30 minutes. The supernatant was removed into a Schlenk flask in glovebox and then titrated with $Te_2Ph_2$ and kept carefully at 4 °C.

**Preparation of Lithium Reagent 4a**

In a dry Schlenk flask charged with argon, the 2,7-dibromide-9,9-dihexylfluorine (**0a**) (147.6 mg, 0.3 mmol) was dissolved in THF (3 mL) and the solution was cooled down to -78 °C. $^{t}$BuLi (0.79 mL, 1.2 mmol, 1.52 M in n-pentane) was added dropwise and the resulted solution was stirred at the same temperature for 0.5 h. After that, solvent was removed by vacuum and the residue of **4a** (0.3 mmol) was ready for next usage.

**Preparation of Lithium Reagent 4c** (**Supplementary Fig. 1**)

To a solution of **A** (2.86 g, 10 mmol) in dichloromethane (100 mL) at 0 °C was added a solution of bromine (3.52 g, 22 mmol) and dichloromethane dropwise. After 12 hours of stirring with temperature allowed warming to room temperature, 30 mL of saturated sodium bisulfite were added and the mixture was stirred for another 30 minutes. The phases were separated and the organic phase was washed two times with 30mL of sodium bisulfite, two times with distilled water, dried over $MgSO_4$ and evaporated to give **B** (4.31 g, 97% isolated yield). Then **B** was dissolved in 50 mL acetone, $K_2CO_3$ (5.53 g, 40 mmol) and 1-Iodododecane (8.88 g, 30 mmol) were added and the mixture was stirred at reflux for 12 hours. After the reaction, 50 mL of dichloromethane and 50 mL of water were added. The solution was washed twice with 50 mL saturated $NaHCO_3$ solution and once with 50 mL brine. The organic phase was dried and evaporated under reduced pressure. The yellow oil was purified by column chromatography on silica gel (Hexane/Ethyl acetate = 99/1) to give the product **C** (6.40 g, 82% isolated yield for two steps). The protocol for lithiation of **C** leading to **4c** is the same as **0a** to **4a**.

## General Procedures for Polycondensations

**Polycondensation between Grignard reagent 1 and dimethoxyarenes 2** (**Supplementary Table. 1**)

In a dry Schlenk flask charged with argon, Grignard reagent **1** (0.3 mmol, THF solution) was added. THF was then removed under vacuum at 0 °C. Toluene (1 mL) was then added, stirred for 5 minutes and again removed under vacuum. After that, dimethoxyarenes **2** (0.3 mmol), **Catalyst A** [$NiCl_2(PCy_3)_2$ (5 mol%) and $PCy_3$ (10 mol%)] or **B** [$Ni(cod)_2$ (5 mol%) and ICy (10 mol%), ICy was *in-situ* prepared by treating ICy • HCl with stoichiometric amounts of EtMgBr] and toluene (1 mL) were added. This reaction mixture was stirred overnight (> 12 h) at room temperature or with heating (**Fig. 2**) before quenched by 1M HCl (5 mL). The aqueous layer was extracted with $CH_2Cl_2$ (3 x 5 mL), dried over $MgSO_4$, filtered, and concentrated under vacuum. The residue was then dissolved in a minimum amount of $CH_2Cl_2$ (ca. 1 mL) and precipitated into MeOH (100 mL). The precipitate was collected and dried under vacuum.

**Polycondensation between organolithium reagent 4 and dimethoxyarenes 2** (**Supplementary Table. 2**)

In a dry Schlenk flask charged with argon, organolithium reagent **4** (0.3 mmol, THF solution) was prepended according to above procedure**.** THF was then removed under vacuum at 0 °C. Toluene (1 mL) was then added, stirred for 1 minute and again removed under vacuum. After that, dimethoxyarenes **2** (0.3 mmol), $Ni(cod)_2$ (0.03 mmol), SIMes (0.03 mmol, SIMes was *in-situ* prepared by treating SIMes • HCl with stoichiometric amounts of EtMgBr) and toluene (1 mL) were added. This reaction mixture was stirred overnight (> 12 h) at 90 °C before quenched by 1M HCl (5 mL). The aqueous layer was extracted with $CH_2Cl_2$ (3 x 5 mL), dried over $MgSO_4$, filtered, and concentrated under vacuum. The residue was then dissolved in a minimum amount of $CH_2Cl_2$ (ca. 1 mL) and precipitated into MeOH (100 mL). The precipitate was collected and dried under vacuum.

**Polycondensation between Grignard reagent 1 and ammonium salts 5** (**Supplementary Table. 3**)

In a dry Schlenk flask charged with argon, Grignard reagent **1** (0.3 mmol, THF solution) was added. Then the ammonium salts **5** (0.3 mmol) and $PdCl_2(PCy_3)_2$ (0.003 mmol) was added in to the above solution of **1**. The resulted mixture was stirred overnight (> 12 h) at room temperature before quenched by water (5 mL). The mixture was extracted with $CH_2Cl_2$ (3 x 5 mL), dried over $MgSO_4$, filtered, and concentrated under vacuum. The resulting residue was then dissolved in a minimum amount of $CH_2Cl_2$ (ca. 1 mL) and precipitated into MeOH (100 mL). The precipitate was collected and dried under vacuum.

**Supplementary Table 1.** Reaction Conditions, Isolation Results and $^{1}$H-NMR Information for Products **3** obtained from Polycondensation between Grignard reagent **1** and Dimethoxyarenes **2**

| Polymer 3 | Conditions | Isolation | $^{1}$H-NMR (500 MHz, $CDCl_3$, 25 °C) |
|---|---|---|---|
| 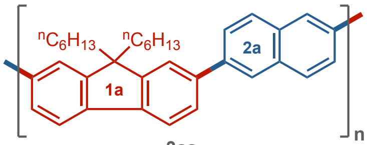 3aa | Catalyst A r.t. | Yield = 96% $M_n$ = 23.2 kDa PDI = 3.02 | δ 7.69-8.19 (m, 12H), 2.21 (br s, 4H), 1.14 (m, 12H), 0.49-0.81 (m, 10H). |
| 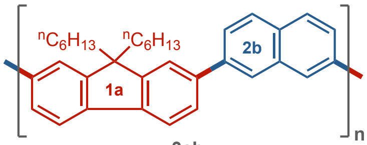 3ab | Catalyst A r.t. | Yield = 96% $M_n$ = 23.6 kDa PDI = 2.73 | δ 7.77-8.19 (m, 12H), 2.39-1.77 (br s, 4H), 1.14 (m, 12H), 0.76-0.81 (m, 10H). |
| 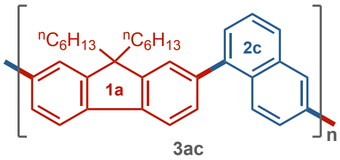 3ac | Catalyst A r.t. | Yield = 82% $M_n$ = 13.2 kDa PDI = 2.53 | δ 7.73-8.32 (m, 12H), 2.19 (br s, 4H), 1.18-1.30 (m, 12H), 0.72-0.98 (m, 10H). |
| 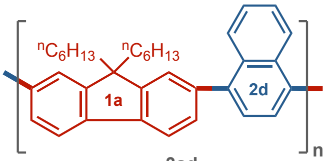 3ad | Catalyst B 70 °C | Yield = 83% $M_n$ = 11.3 kDa PDI = 2.28 | δ 7.19-8.13 (m, 12H), 2.37 (br s, 4H), 1.05-1.28 (m, 12H), 0.76-0.92 (m, 10H). |
| 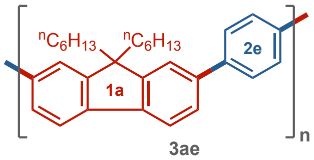 3ae | Catalyst B 70 °C | Yield = 88% $M_n$ = 12.1 kDa PDI = 2.32 | δ 7.66-7.82 (m, 10H), 2.10 (br s, 4H), 1.11 (m, 12H), 0.76-0.80 (m, 10H). |
| 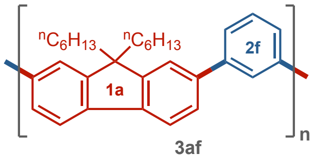 3af | Catalyst B 70 °C | Yield = 86% $M_n$ = 13.6 kDa PDI = 2.56 | δ 7.26-7.97 (m, 10H), 2.09 (br s, 4H), 1.01-1 (m, 12H), 0.77 (m, 10H). |
| 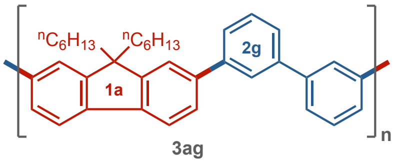 3ag | Catalyst B 120 °C | Yield = 93% $M_n$ = 12.7 kDa PDI = 2.41 | δ 7.11-7.84 (m, 14H), 2.13 (br s, 4H), 1.29-1.33 (m, 12H), 0.80-0.89 (m, 10H). |
| 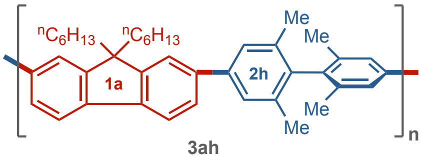 3ah | Catalyst B 70 °C | Yield = 80% $M_n$ = 14.3 kDa PDI = 2.33 | δ 7.46-7.82 (m, 10H), 1.94-2.10 (m, 15H), 1.11-1.14 (m, 12H), 0.74-0.79 (m, 10H). |

| | | | |
|---|---|---|---|
| 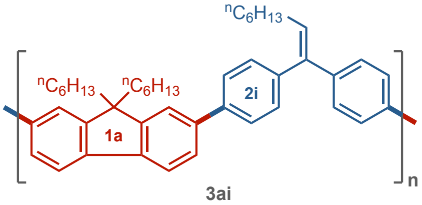 **3ai** | Catalyst B<br>70 °C | Yield = 82%<br>$M_n$ = 9.2 kDa<br>PDI = 2.11 | δ 7.56-7.78 (m, 8H), 7.31-7.40 (m, 4H), 3.86 (d, J = 1.8 Hz, 0H), 2.24 (br s, 1H), 2.00-2.06 (br s, 4H), 1.29-1.51 (m, 8H), 1.06-1.14 (m, 13H), 0.87-0.90 (m, 3H), 0.74-0.77 (m, 10H). |
| 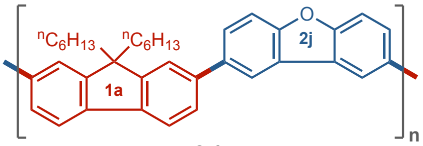 **3aj** | Catalyst B<br>70 °C | Yield = 93%<br>$M_n$ = 10.6 kDa<br>PDI = 2.29 | δ 7.70-7.85 (m, 12H), 2.14 (m, 4H), 1.14 (m, 12H), 0.80 (m, 10H) |
| 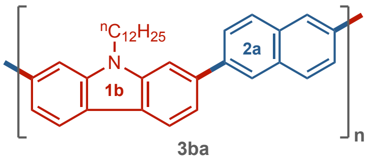 **3ba** | Catalyst A<br>r.t. | Yield = 92%<br>$M_n$ = 17.9 kDa<br>PDI = 2.58 | δ 7.69-8.25 (m, 12H), 0.86-2.03 (m, 25H). |

**Supplementary Table 2.** Reaction Conditions, Isolation Results and $^1$H-NMR Information for Products **3** obtained from Polycondensation between organolithium reagents **4** and dimethoxyarenes **2**

| Polymer 3 | Isolation | $^1$H-NMR (500 MHz, $CDCl_3$, 25 °C) |
|---|---|---|
| 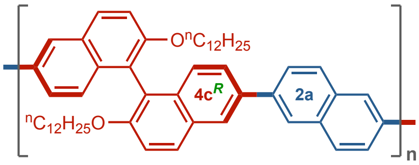 **3c$^R$a** | Yield = 89%<br>$M_n$ = 12.3 kDa<br>PDI = 2.36 | δ 7.26-8.22 (m, 16H), 3.94 (br s, 4H), 0.84-1.43 (m, 46H). |
| 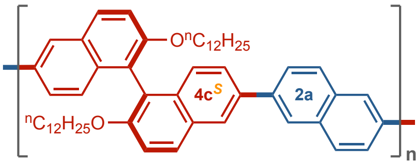 **3c$^S$a** | Yield = 84%<br>$M_n$ = 13.1 kDa<br>PDI = 2.42 | δ 7.26-8.22 (m, 16H), 3.98 (br s, 4H), 0.85-1.43 (m, 46H). |

**Supplementary Table 3.** Reaction Conditions, Isolation Results and $^{1}$H-NMR Information for Products **3** obtained from Polycondensation between Grignard reagents **1** and ammonium salts **5**

| Polymer 3 | Isolation | $^{1}$H-NMR (500 MHz, $CDCl_3$, 25 °C) |
|---|---|---|
| 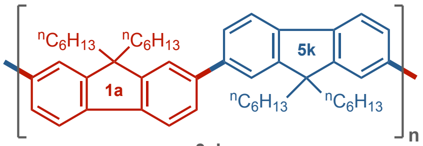  **3ak** | Yield = 95%<br>$M_n$ = 23.4 kDa<br>PDI = 2.64 | δ 7.68-7.86 (m, 6H), 2.13 (br s, 4H), 1.14-1.19 (m, 12H), 0.76-0.85 (m, 10H). |
| 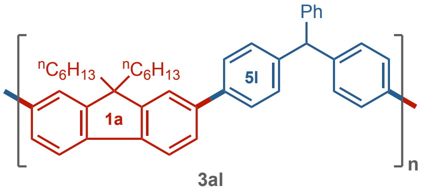  **3al** | Yield = 88%<br>$M_n$ = 24.0 kDa<br>PDI = 2.51 | δ 7.56-7.76 (m, 10H), 7.24-7.34 (m, 9H), 5.67 (s, 1H), 2.01 (br s, 4H), 1.04-1.12 (m, 12H), 0.71-0.77 (m, 10H). |
| 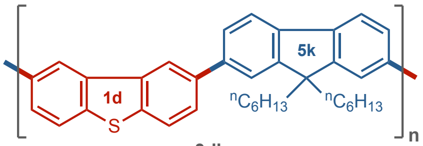  **3dk** | Yield = 84%<br>$M_n$ = 13.5 kDa<br>PDI = 2.48 | δ 7.50-8.55 (m, 12H), 2.14 (br s, 4H), 1.11-1.21 (m, 12H), 0.76-0.81 (m, 10H). |

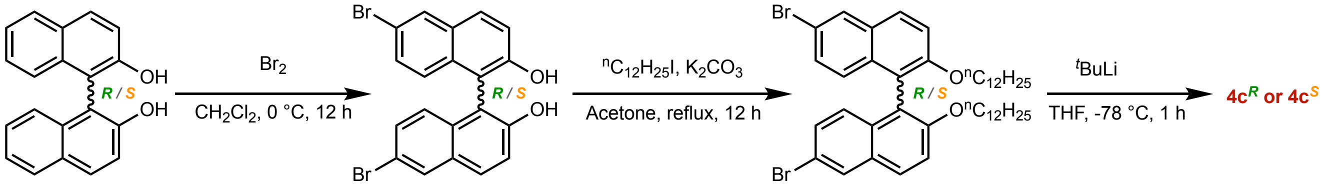


**A** **B** **C**

**Supplementary Figure 1.** Preparation of Lithium Reagent **4c**

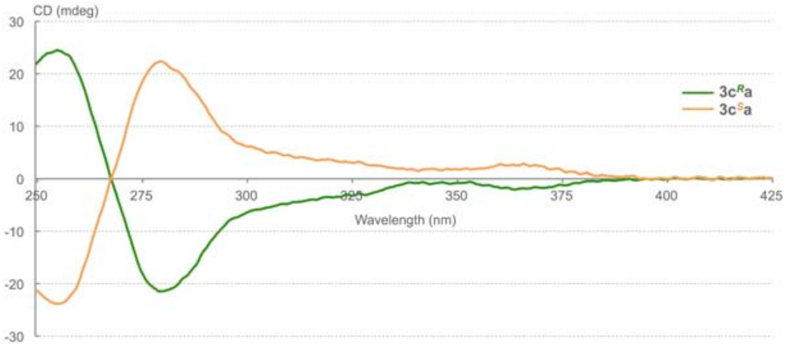


**Supplementary Figure 2.** Circular dichroism (CD) spectra of **$3c^R$a** and **$3c^S$a**. Such result indicates that no racemization occurred at the axially asymmetric positions in BINOL moiety.

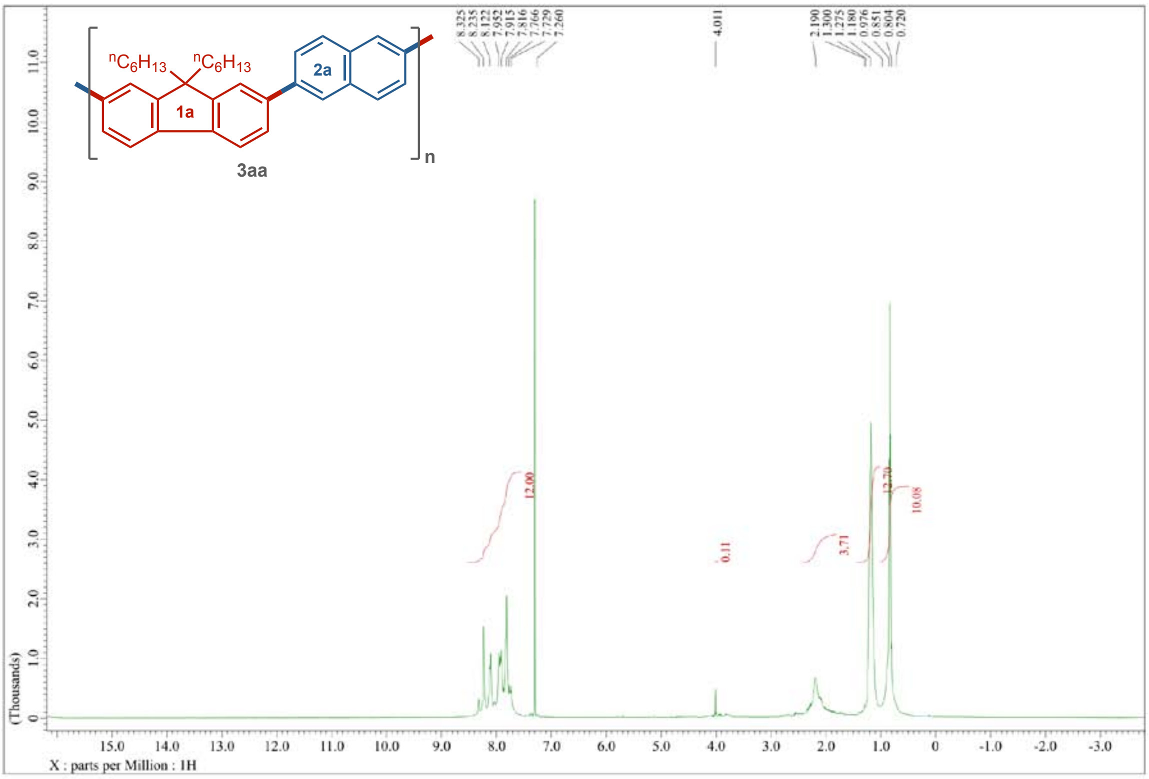


**Supplementary Figure 3.** $^1$H NMR Spectrum of **3aa**

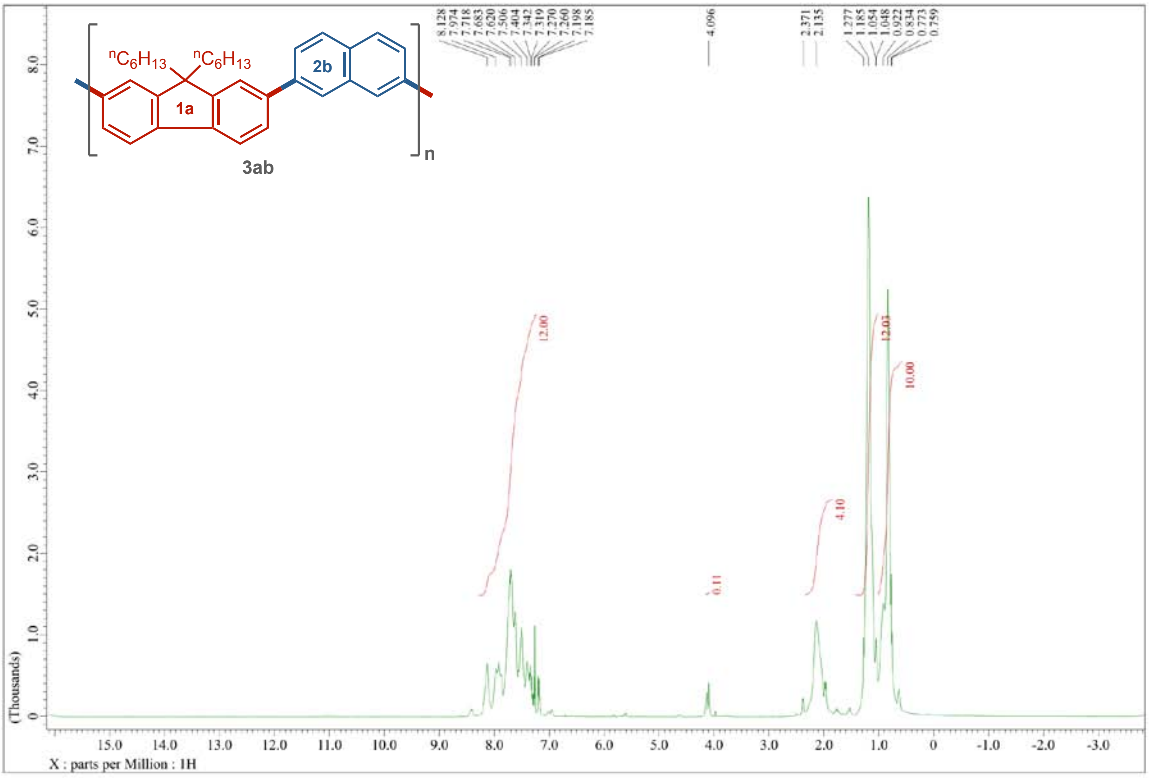


**Supplementary Figure 4.** $^1$H NMR Spectrum of **3ab**

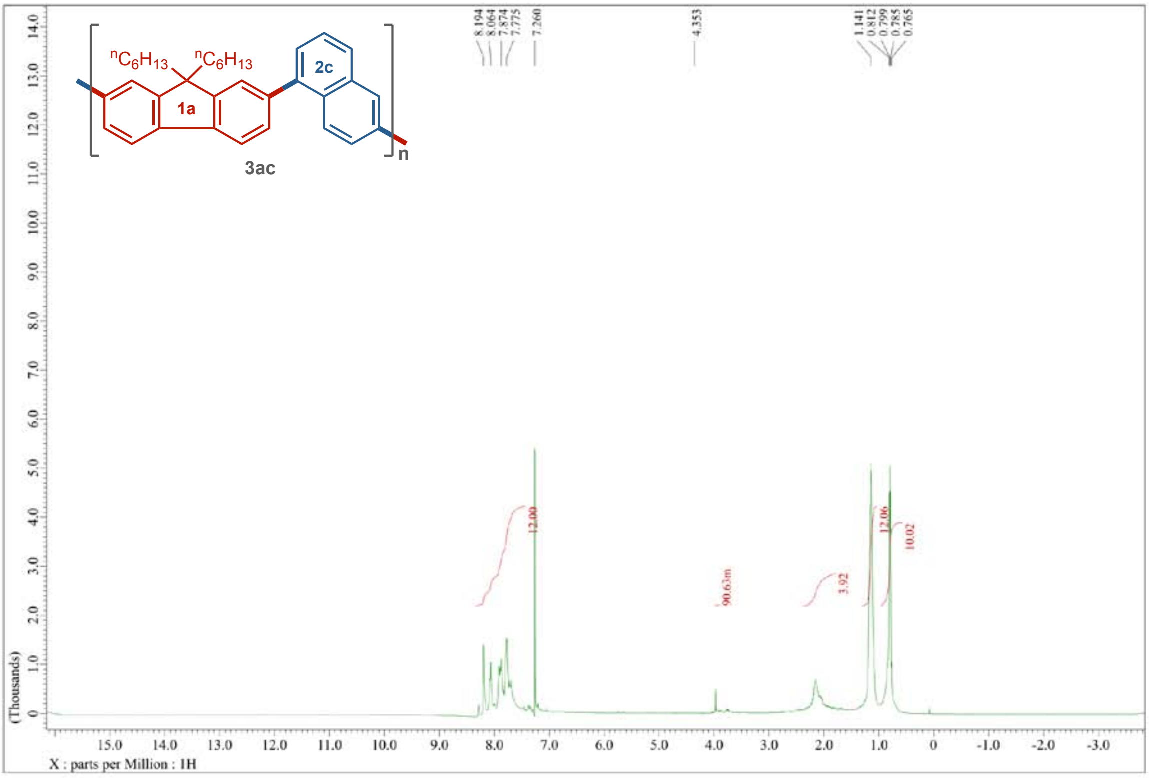


**Supplementary Figure 5.** $^{1}$H NMR Spectrum of **3ac**

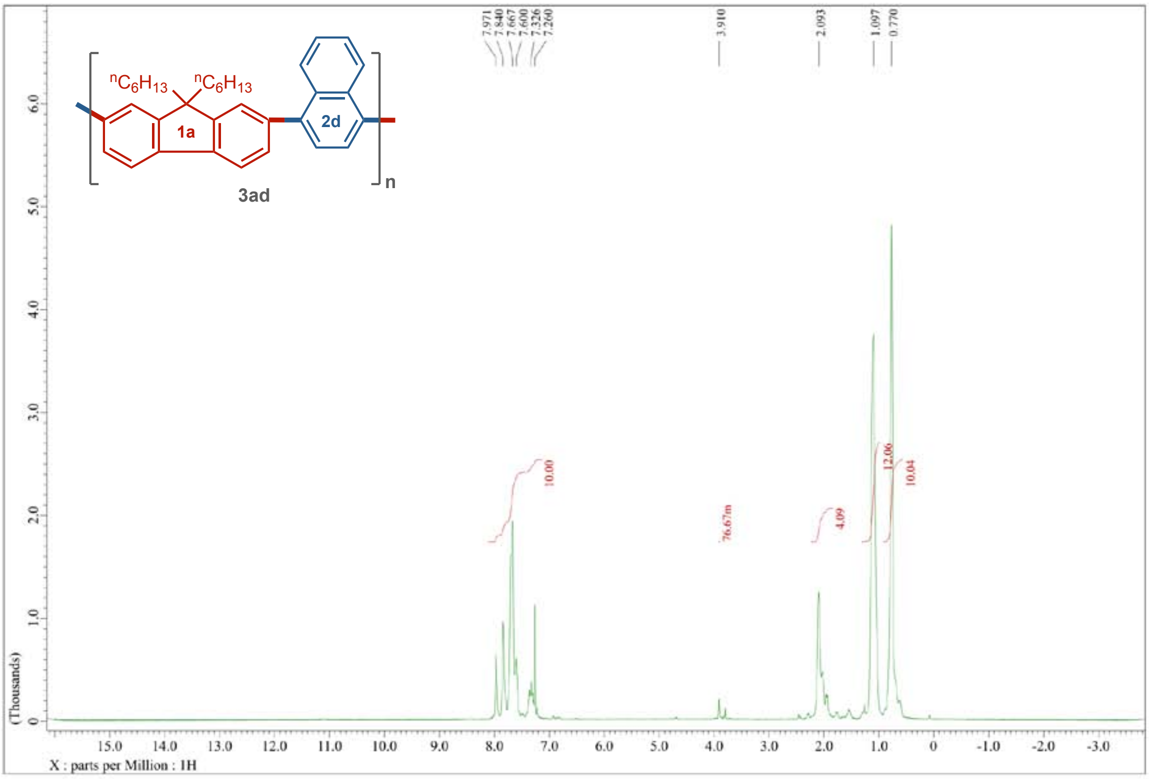


**Supplementary Figure 6.** $^{1}$H NMR Spectrum of **3ad**

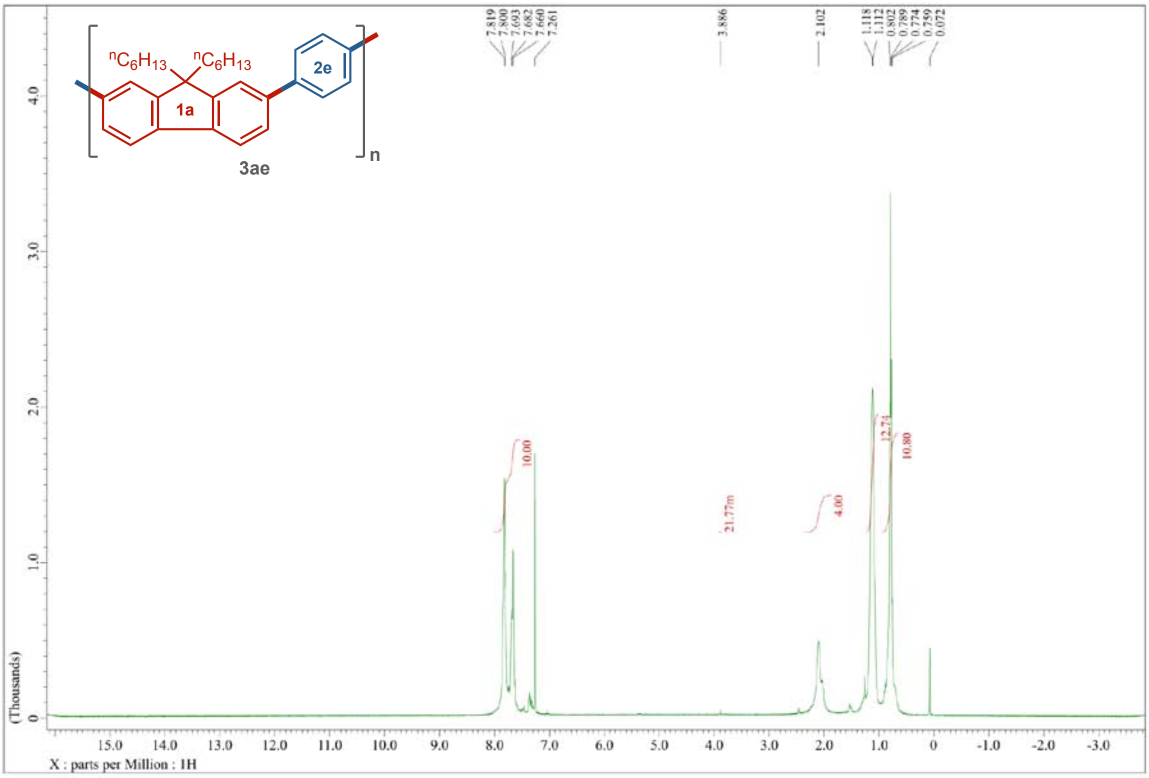


**Supplementary Figure 7.** $^1$H NMR Spectrum of **3ae**

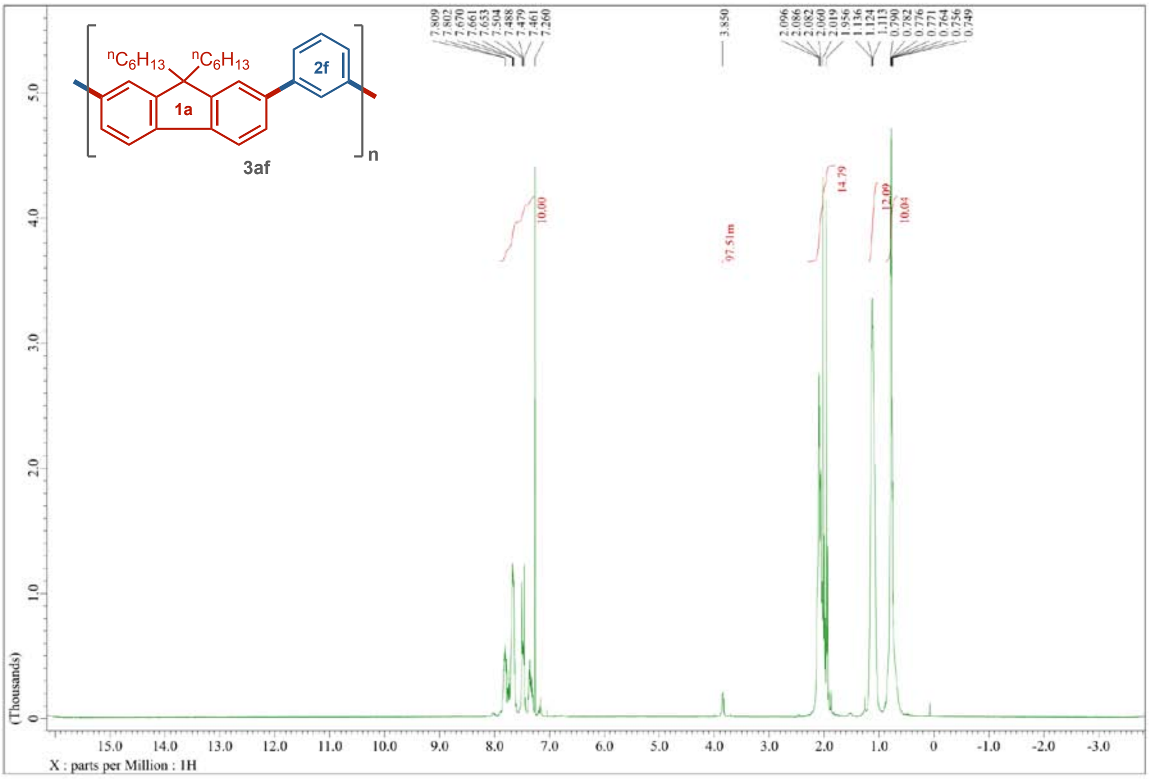


**Supplementary Figure 8.** $^1$H NMR Spectrum of **3af**

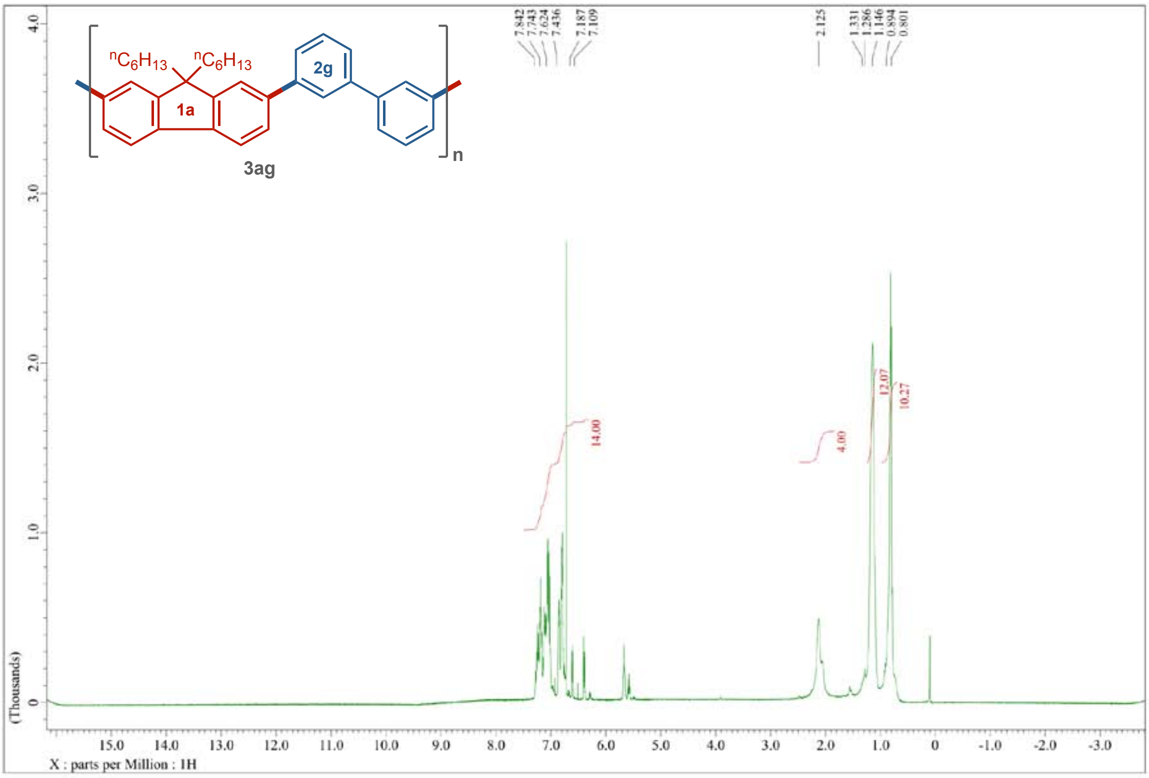


**Supplementary Figure 9.** $^{1}$H NMR Spectrum of **3ag**

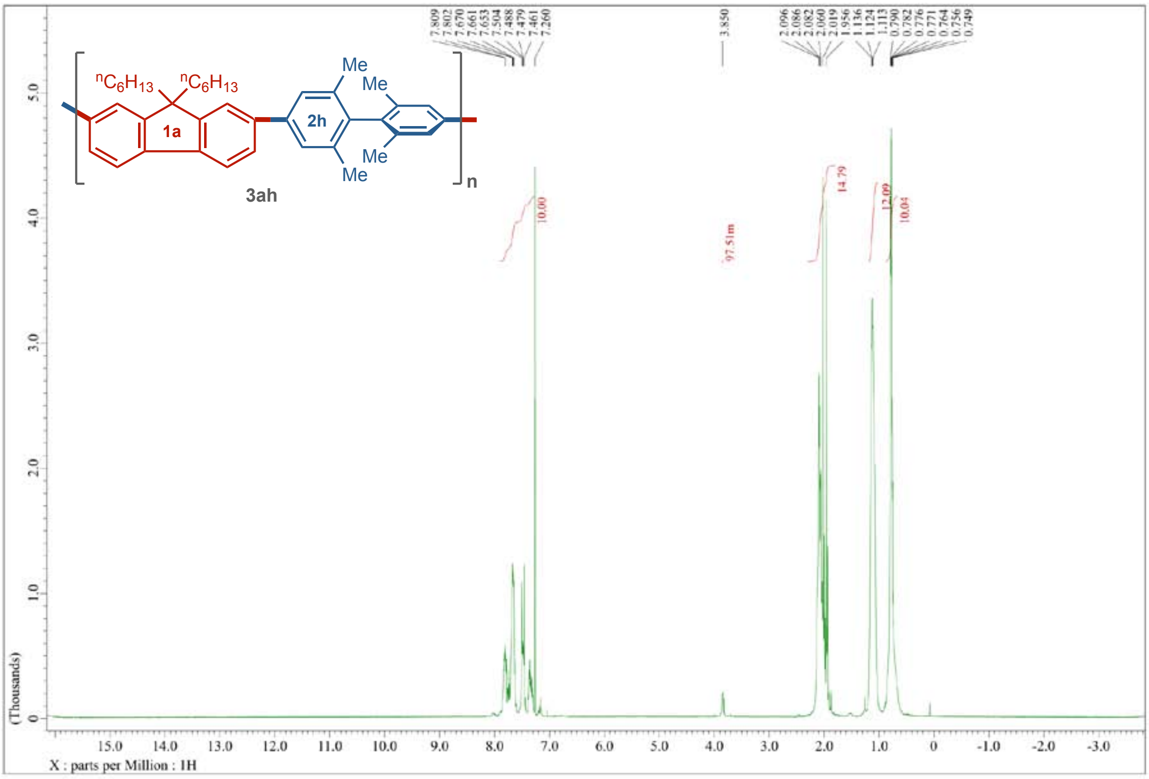


**Supplementary Figure 10.** $^{1}$H NMR Spectrum of **3ah**

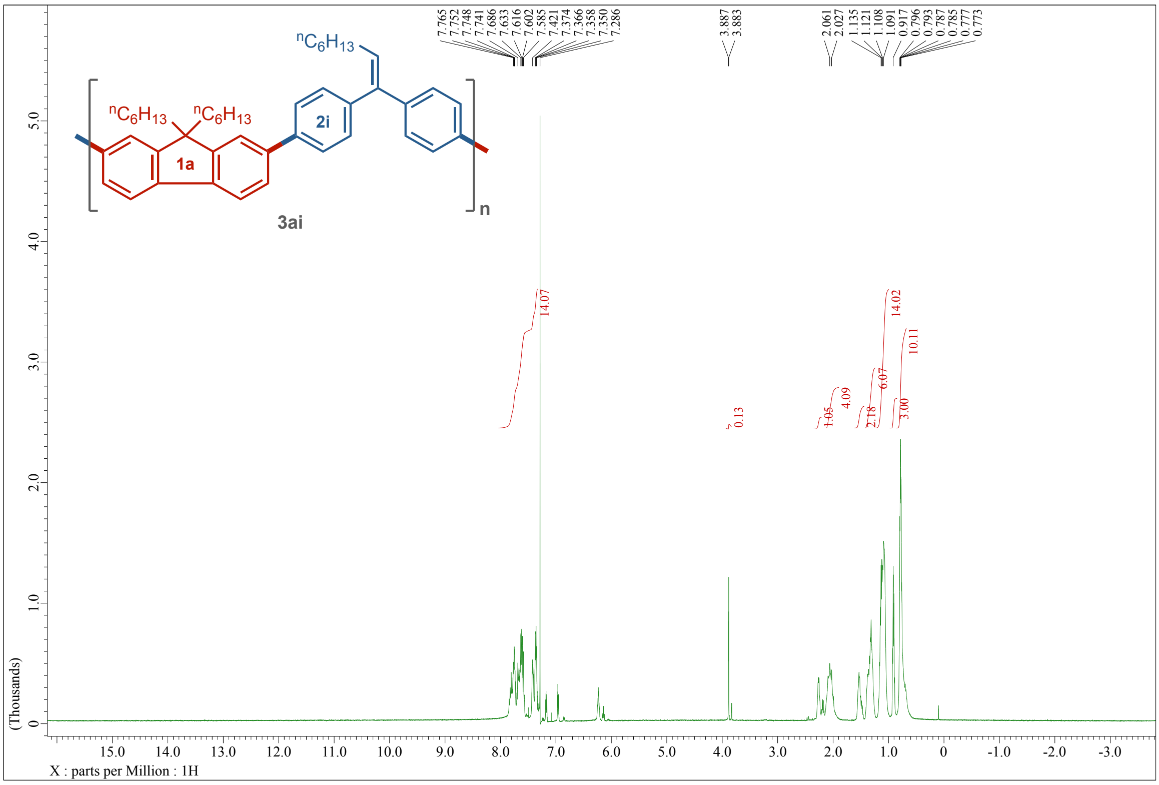


**Supplementary Figure 11.** $^{1}$H NMR Spectrum of **3ai**

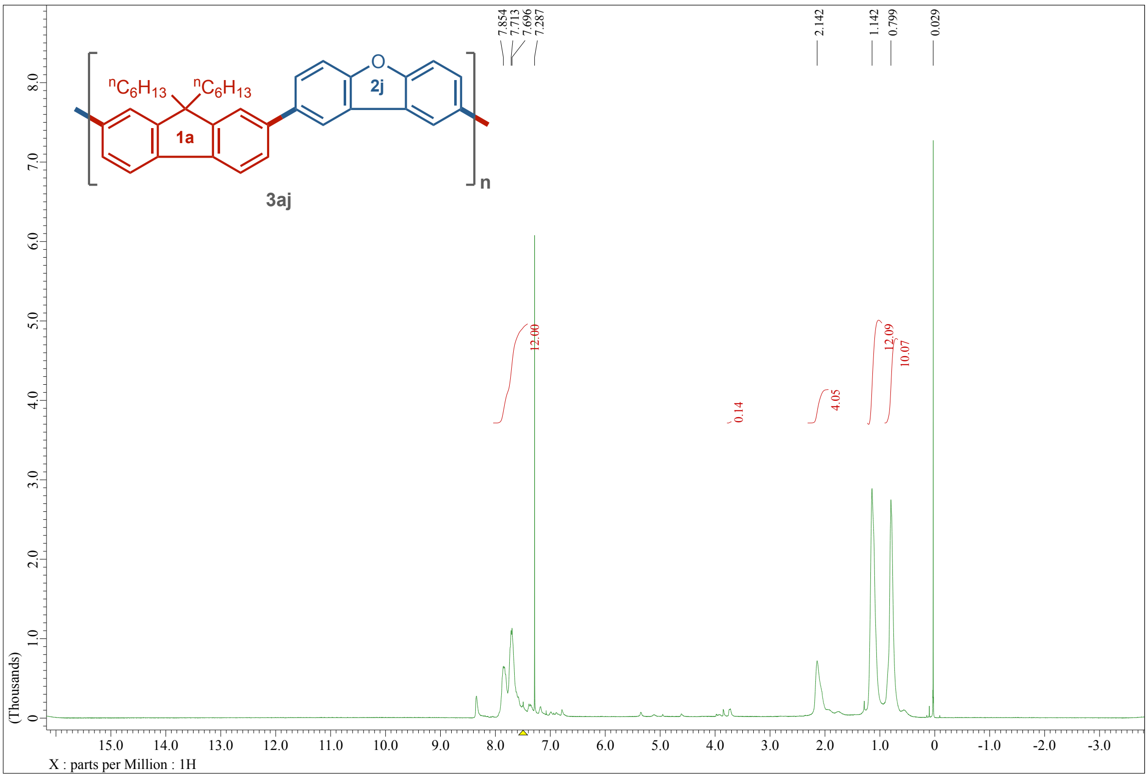


**Supplementary Figure 12.** $^{1}$H NMR Spectrum of **3aj**

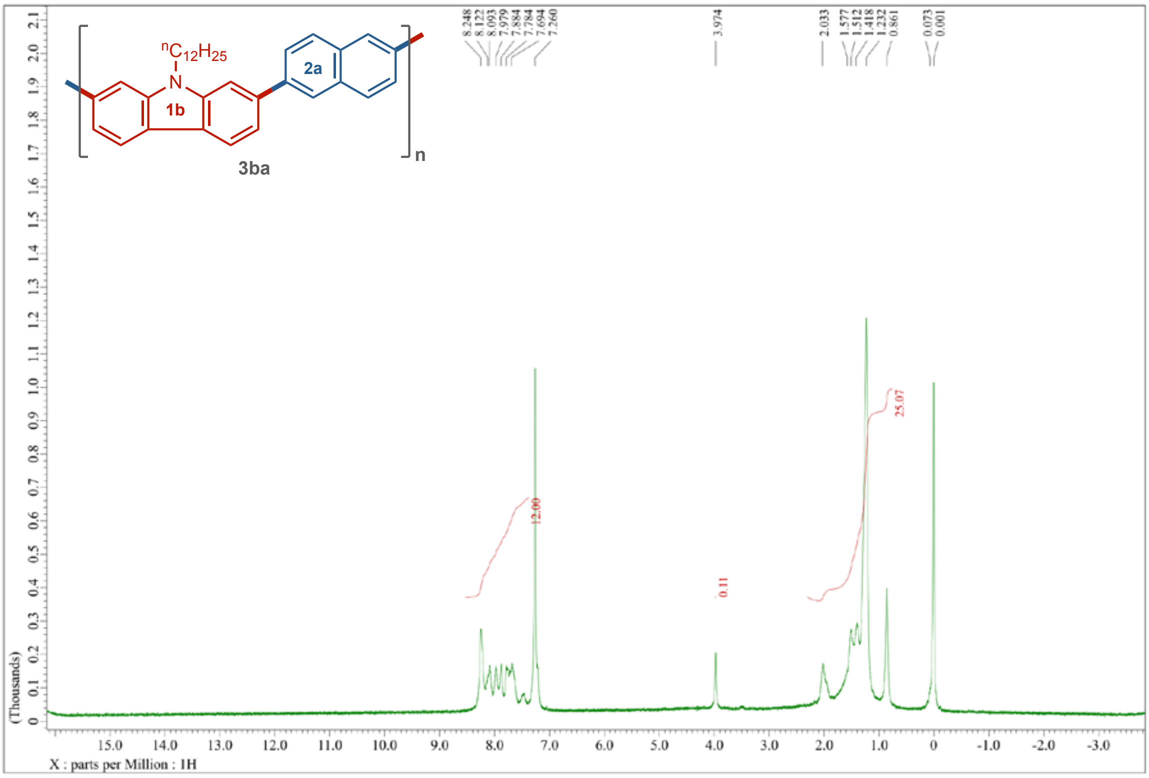


**Supplementary Figure 13.** $^1$H NMR Spectrum of **3ba**

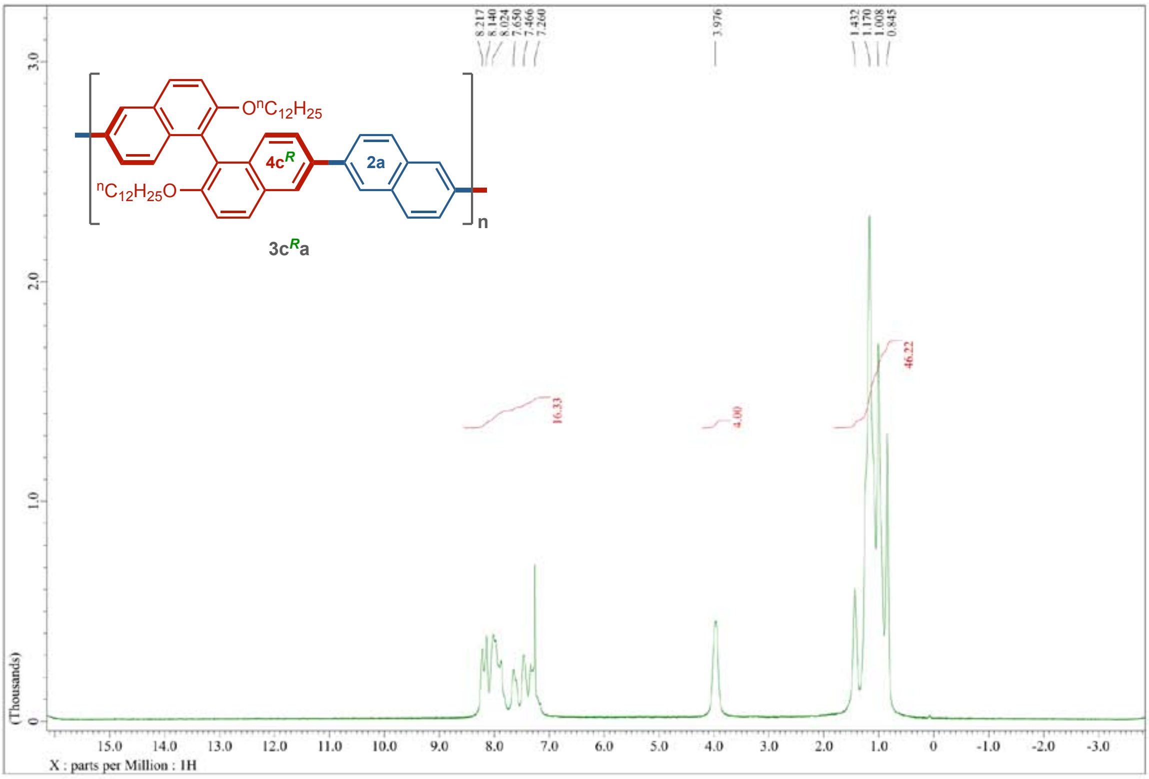


**Supplementary Figure 14.** $^1$H NMR Spectrum of **3c$^R$a**

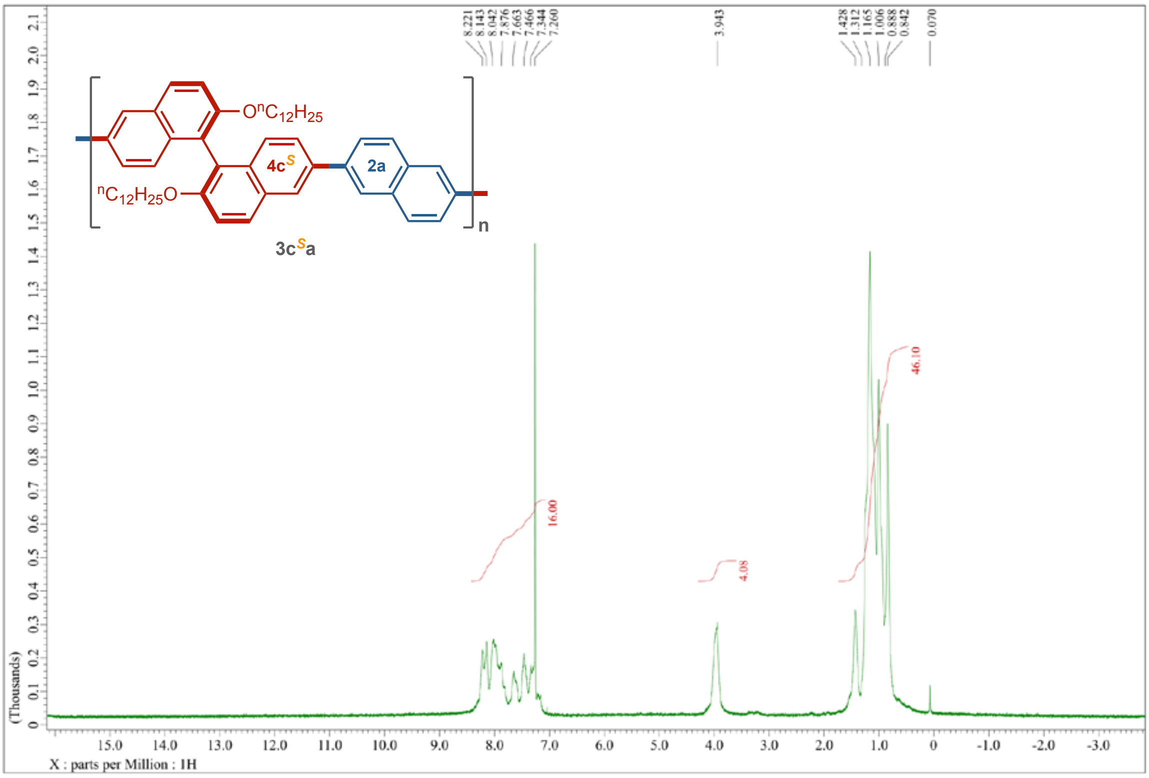

Supplementary Figure 15. $^1$H NMR Spectrum of **3c$^S$a**

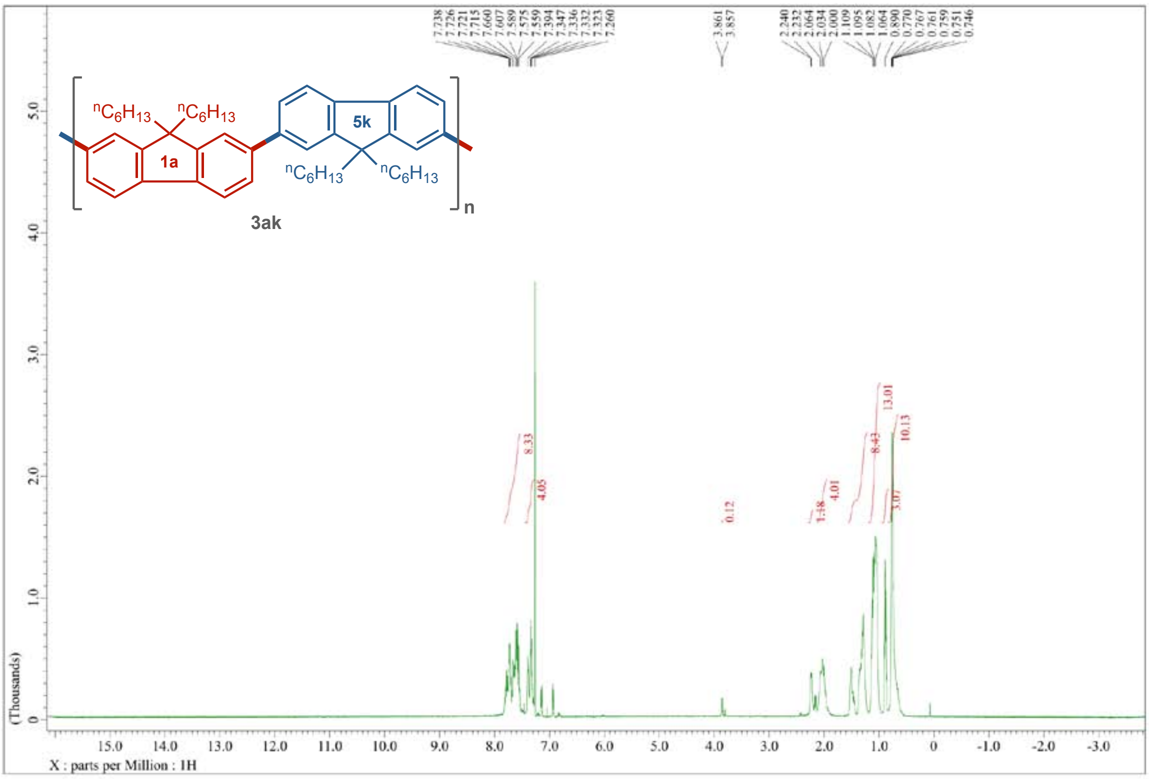

Supplementary Figure 16. $^1$H NMR Spectrum of **3ak**

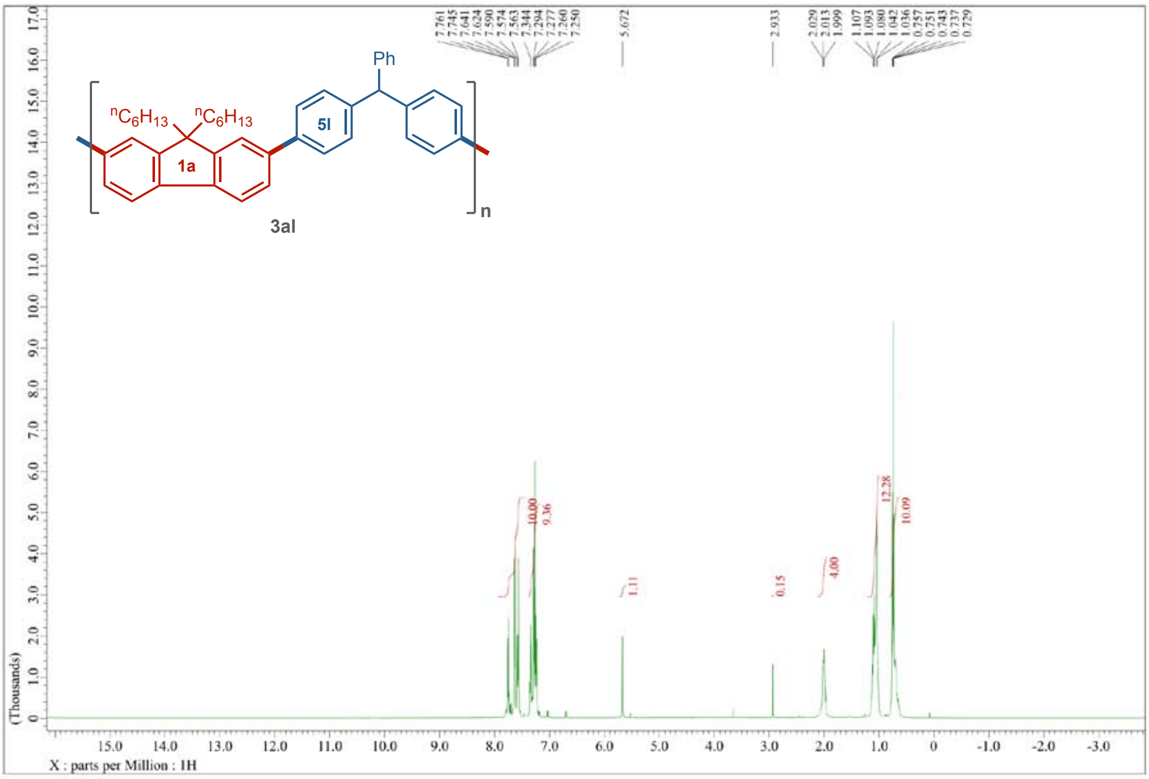

**Supplementary Figure 17.** $^1$H NMR Spectrum of **3al**

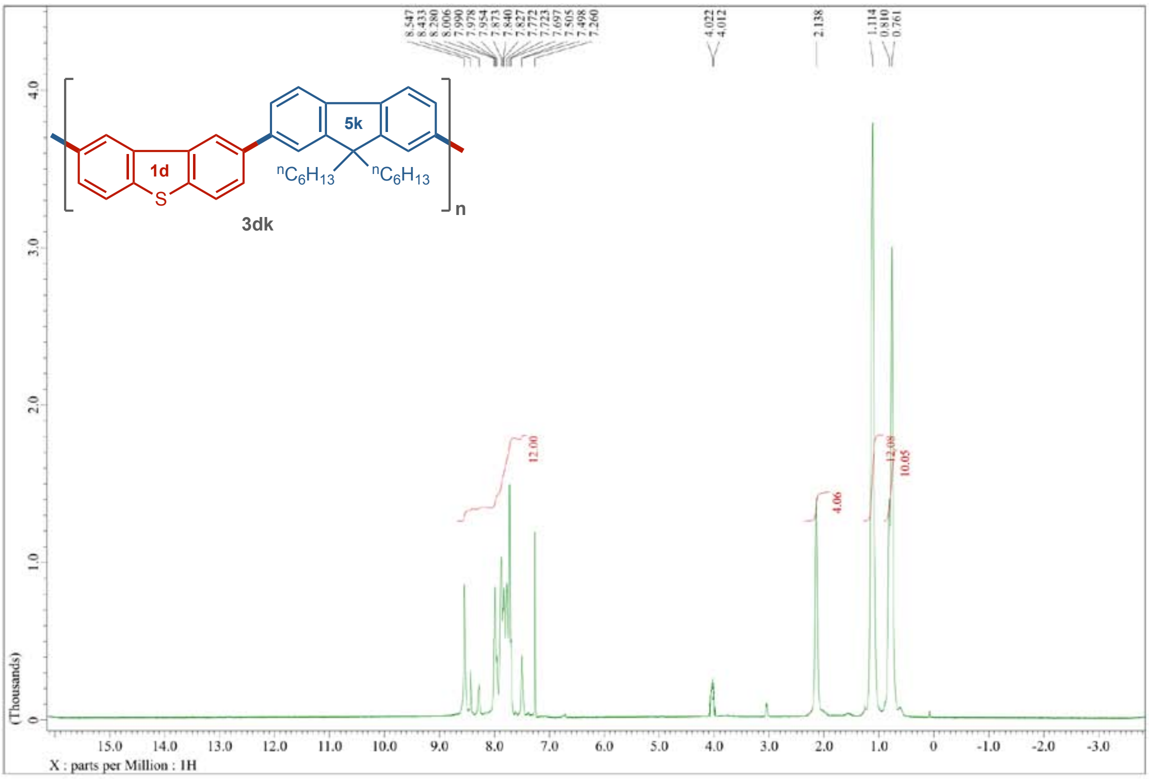

**Supplementary Figure 18.** $^1$H NMR Spectrum of **3dk**
